# Understanding ADA2 and the etiology of DADA2: connecting the loose ends

**DOI:** 10.3389/fmed.2026.1918794

**Published:** 2026-10-28

**Authors:** Ole Kristian Greiner-Tollersrud

**Affiliations:** Lysosomal Research group, IMB, Health Faculty, UiT the Arctic University of Norway, Tromsø, Norway

**Keywords:** autoinflammation, DADA2, immunodeficiency, lysosomal, NET, N-glycan

## Abstract

Adenosine deaminase 2 (ADA2) deficiency (DADA2) is a recently discovered autosomal recessive immunodeficiency primarily affecting the bone marrow and vascular system. Previously ADA2 was regarded as a secretory glycoprotein downregulating inflammation by deaminating adenosine and functioning as a growth factor of immune cells. An alternative model was recently proposed suggesting that ADA2 binds and modifies oligonucleotides within the lysosomes. A lysosomal role is not easily reconciled with the increased secretion of ADA2 during inflammation, such as for example tuberculosis, suggesting that ADA2 has an immunoregulatory role in plasma. Here we present a new model where ADA2 may be stored in lysosome-like vesicles after sorting into the lysosomal pathway and released by exocytosis upon signaling. Thus, ADA2 may serve two roles, in the extracellular space contributing to targeting and clearance of oligonucleotide complexes and in the lysosomes taking part in the oligonucleotide metabolism.

## Graphical Abstract

**Figure:**
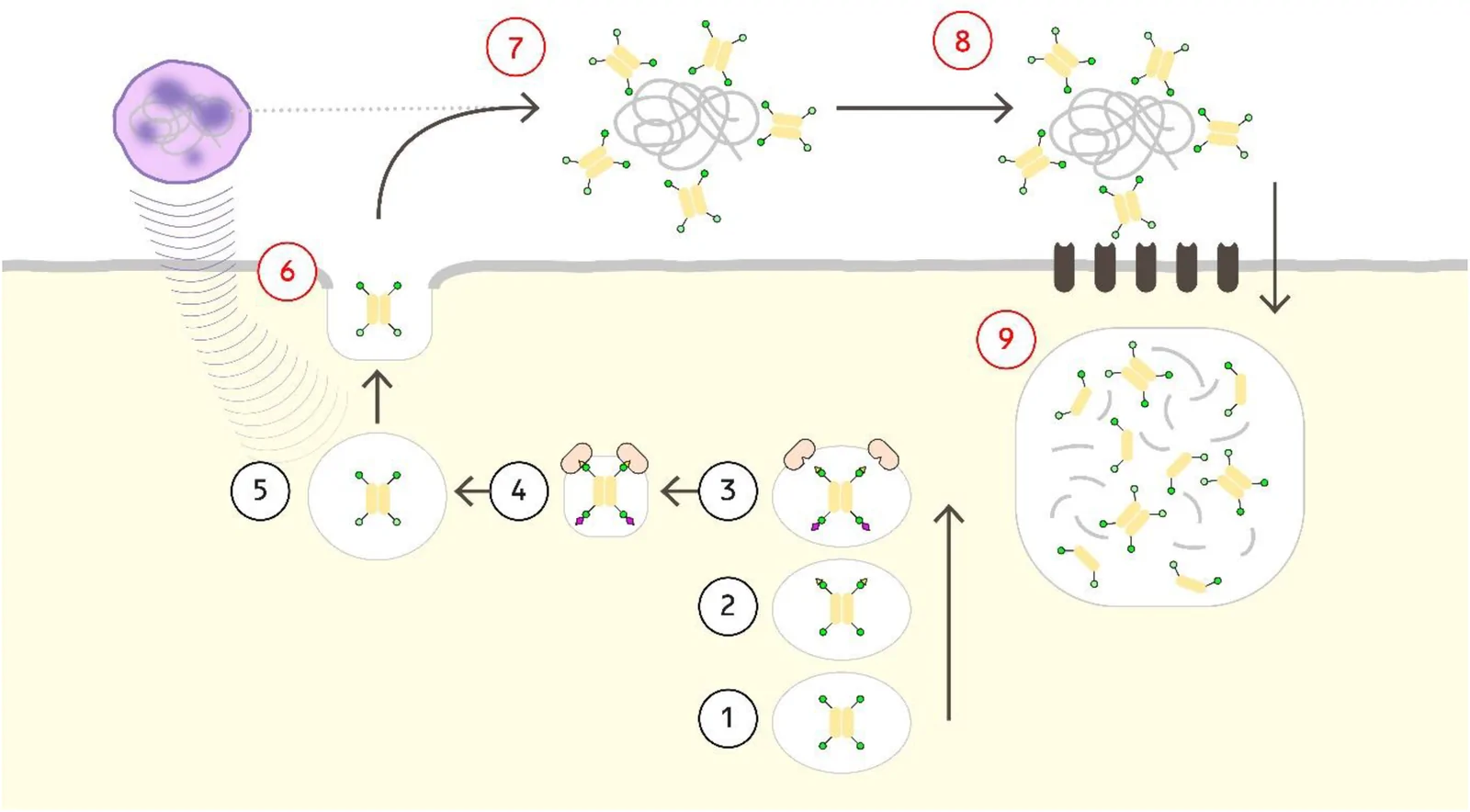
1. ADA2 is synthesized as an N-glycosylated homodimer in endoplasmic reticulum. 2. In cis-Golgi phosphate groups are added to selected N-glycans. 3.The phosphorylated glycans are recognized by the mannose-6-phosphate receptor (MPR) in trans Golgi, while the unphosphorylated glycans are processed into complex structures. 4. The MPR-ADA2 complex is transported within vesicles towards the endolysosomal system. 5. ADA2 is released from MPR in late endosomes and acid exoglycosidases trim N-glycans into mannose-terminal structures. 6. From late endosomes ADA2 may be sorted into secretory lysosomes that are subjected to exocytosis upon danger signals. 7. ADA2 may bind complexes of oligonucleotides in plasma. 8.The mannose receptor may recognize mannose-terminal N-glycan structures of ADA2 facilitating the transport of the complex into phagolysosomes, where 9. ADA2 may take part in the lysosomal oligonucleotide metabolism. Step 6-9 are labeled with red color as they have not been experimentally verified. Sugar symbols: 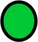 high mannose; 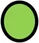 low mannose; 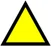 mannose-6-phosphate; 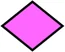 complex

## Introduction

Almost 50 years ago a residual adenosine deaminase (ADA) activity was discovered in patients with severe combined immunodeficiency (SCID) due to loss of function mutations in the gene encoding for human adenosine deaminase (ADA1) (1) The residual ADA activity (hereafter named ADA2) was a different gene product than ADA1 as the potent ADA1 inhibitor *erythro*-9-(2-hydroxyl-3-nonyl) adenine (EHNA), had little effect on ADA2 (1, 2). Moreover, ADA2 differed from ADA1 by exhibiting a 100 fold higher K_m_ value against adenosine and being inactive against adenine and AMP (2). Moreover, ADA2 is a homodimer of 110 kDa, while ADA1 a monomer of 40 kDa, and ADA2 has a higher heat stability (2). In 1996 an insect derived growth hormone was isolated from conditioned media of an embryonal cell line of the flesh fly *Sarcophaga peregrina*. It was a protein of 100 kDa consisting of identical subunits of 50 kDa and was named Insect-Derived Growth Factor (IDGF) (3). Shortly after the deduced amino acid sequence derived from the Cat Eye syndrome Critical Region 1 (CECR1) gene was identified as a homologue of IDGF (4). Human ADA2 was first purified from a commercial IgG fraction which revealed that the residual ADA activity in SCID patients originated from a protein containing an amino acid sequence identical to the deduced amino acid sequence of CECR1 (5). Based on the studies on insect IDGF (also named ADGF) (3) it was suggested that ADA2 is a putative growth factor with adenosine deaminase activity (5). When the structure of ADA2 was determined, a protruding IgG like domain was identified and proposed to contain the binding site to a yet unknown growth factor receptor, that accordingly was named “putative receptor binding” (prb) domain (6). To date, however, no specific receptor for ADA2 has been identified and validated, nor has a receptor-dependent signaling pathway been demonstrated. Since IDGF stimulated cell proliferation in *Drosophila* by depleting extracellular adenosine (7, 8), IDGF may not be a growth factor by itself, but a regulator of growth by depleting the extracellular concentration of the growth inhibiting factor, adenosine (9). The interest in ADA2 within human immunology was sparked by three independent reports in 2014 on patients affected by a deficiency of ADA2 (DADA2) (10–12). DADA2 is an autosomal recessive systemic immunodeficiency, primarily affecting the bone marrow and vascular systems. Vasculitis manifests in early childhood as livedo racemosa, early-onset ischemic strokes and systemic polyarteritis nodosa. The effect on bone marrow may result in mild to severe cytopenia of all lineages (anemia, lymphopenia and thrombocytopenia) (10–20). Bone marrow transplantation appears to be a curative treatment, conforming to the main expression of ADA2 in monocytes/macrophages (21, 22). There is no causal therapy for the treatment of DADA2, as plasma replacement therapy did not give durable results (23). Tumor Necrosis Factor alpha (TNF) antagonists are currently regarded for first-line treatment of the vasculitis phenotype (23, 24), but with limited therapeutic effect in patients with severe bone marrow involvement (25). Presently there is no consensus regarding the function of ADA2 and the molecular pathophysiology of DADA2, which has limited the development of a targeted therapy. Our aim is to present a model of the ADA2 function which conforms to the pathophysiology of DADA2 and can thus be used as a framework for future research.

## The physiological substrate of ADA2

As presented in Table 1, the affinity of ADA2 towards free adenosine decreased during evolution, as judged from a 100-fold increase of Km from insect to human. This evolution suggested that the physiological substrate of human ADA2 may not be free adenosine. It could be a macromolecule with high affinity to a region in ADA2 different from the active site pocket, as for example the prb-domain (6), containing an adenosine-residue positioned to enter the active site pocket only after formation of the complex (Figure 1). Until recently the prevailing view was that human ADA2 possessed binding sites for two different molecules, the prb domain binding to growth factor receptors and the adenosine binding site binding and hydrolyzing free adenosine (5). This model may apply for insect ADA2 due to its high affinity to free adenosine (Table 1). An argument of ADA2 as a regulator of free plasma adenosine is that the adenosine concentration is significantly increased in plasma of DADA2 patients, from an average of 0.2 *μ*M to 0.4 *μ*M (26). As neutrophils are activated by adenosine *in vitro*, it was inferred that the increase of plasma adenosine in DADA2 might have caused this activation, initiating events leading to the vasculitis phenotype (26). Although ADA2 reversed the adenosine mediated activation in cell cultures, the absence of a similar reversal by ADA1 (26) suggested that it was not a result of the adenosine deaminase activity of ADA2. ADA2 may prevent adenosine from activating neutrophils by binding to adenosine receptors and proteoglycans (27, 28). A direct role of ADA2 in downregulating free adenosine in plasma was suggested by Dhanwani et al. (29). Focusing on interferon production in monocytes and endothelial cells they identified a novel pathway where excess extracellular deoxyadenosine triggered activation of cytosolic dsRNA sensors inducing IFN-*β*. Although the authors acknowledged that this model required an adenosine deaminase with a lower Km than reported for human ADA2 (29) (Table 1) no experimental evidence has been presented that human ADA2 possesses a lower Km in plasma. Therefore, it has been suggested that the high affinity equilibrative nucleoside transporter of erythrocytes (30) is the main route for clearance of free plasma adenosine (9).

**Table 1:** Properties of ADA2 from different species.

Specie	Source	Km Ado(mM)	Kcat (s^−1^)	pH optimum	Native mass
Insect	Lutzomyia longipalpis^h^	0.015	nd	6.5	52
Insect	*Sarcophaga peregrina* ^g^	0.05	170	nd	100
Mollusc	Helix pomatia * ^i^	0.025	170	nd	nd
Frog	*Xenopus laevis A6 cells-1* ^b^	0.26	nd	nd	nd
Frog	*Xenopus laevis A6 cells-2^b^*	0.23	nd	nd	nd
Chicken	Liver^f^	0.14	nd	6.5	100
Human	Spleen^e^	2.0	nd	6.5	92
Human	Serum^d^	2.8	nd	nd	98
Human	Serum^a^	2.5	88	6.5	nd
Human	B-lymphopblasts^c^	3.1	nd	5.5–6	110

Kinetic constants, pH-optimum and native mass of ADA2 from different species. ^a^(5); ^b^(31); ^c^(32); ^d^(33); ^e^(1); ^f^(34); ^g^(7); ^h^(35); ^i^(36). *AMP deaminase activity, nd, not determined

**Figure 1:**
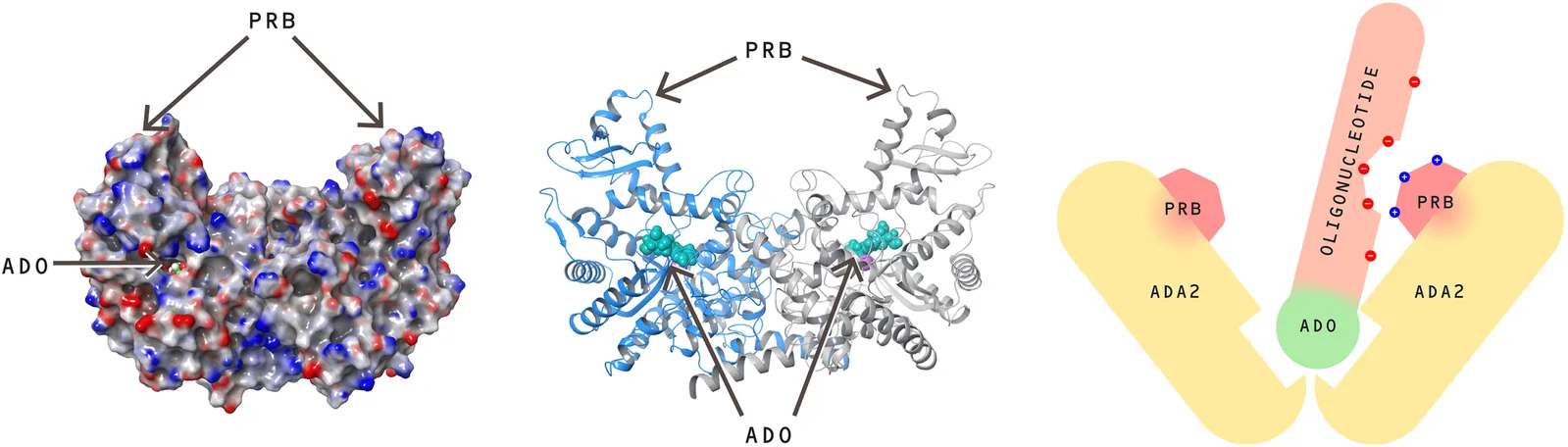
Schematic drawing of an oligonucleotide strand binding to one of the monomers of the ADA2 homodimer (right side). The negatively charged oligonucleotide binds to the positively charged PRB-domain in ADA2. A terminal adenosine (ADO) in the oligonucleotide will access the active site pocket and subsequently hydrolyze into inosine (right side). The two PRB-domains forms the wings of the ADA2 homodimer butterfly structure (left and middle) while the active site pockets are located on the bottom part of the groove formed by two monomers, close to the dimer interface (left and middle). The active sites are located on opposite sides of the homodimer structure which may allow for simultaneous binding of two oligonucleotide strands (left and right). The charge distribution around the PRB-domain and active site is highly positive (left side) which may facilitate electrostatic interactions upon binding between the oligonucleotide strand and ADA2. The molecular models (left and right) are according to the PDB identifier: 3lgg.

ADA2 may modulate the immune response by binding different subsets of immune cells and thus initiating differentiation including macrophage proliferation (27, 28). Cocultures of monocytes from DADA2 patients or ADA2 knock out monocytic cell lines caused disruption of endothelial cell monolayers, likely due to differentiation into M1 proinflammatory type macrophages (10). This appears as an intrinsic event as monocytes with knock out of ADA2 are sufficient to induce a proinflammatory gene expression (37). It is unclear if this M1/M2 polarization switch is due to the binding of ADA2 to a growth factor on macrophages, since such a growth factor has not been identified.

A one-substrate model of ADA2 may explain the butterfly structure of ADA2 (Figure 1) as the prb-domain forms the wings with high affinity to its substrate and the active site pocket located close to the homodimer interface region positioned to bind an optimally located adenosine residue of the substrate (Figure 1). In contrast, ADA1 which only possesses an adenosine deaminase activity, lacks both the prb-domain and the dimer interface (6). Molecular modelling and experimental studies have recently shown that ADA2 can bind oligonucleotides (38, 39) so that their terminal adenosines are hydrolyzed into inosines (38). The dimer interface restricts the binding to only terminal ends of oligonucleotide strands (Figure 1), conferring specificity. The prb-domain is characterized by a high pI, ensuring a positive charge even at neutral pH and allowing for electrostatic interaction with negatively charged oligonucleotide substrate (Figure 1). It has been suggested that porcine brain ADA2 is an acid DNase (40), which is not compatible with a homodimer structure as the interface region prevents binding to endogenous regions of oligonucleotides (Figure 1). The DNase activity may be due to a monomer form of ADA2 (personal communication). However, more experiments are required to exclude a DNase contaminant in the purified ADA2 fraction. A role of ADA2 in the intracellular oligonucleotide metabolism (38, 39) offers an explanation on the interferonopathy of DADA2 (41, 42), as abnormalities of the intracellular oligonucleotide metabolism is often related to increased IFN production. Further experiments are needed to confirm this hypothesis.

## ADA2 is a secretory and a lysosomal protein

ADA2 isolated from porcine brain contains mannose-6-phosphorylated N-glycan structures mainly linked to N122 and N375 and lysosomally trimmed N-glycans (Figure 2) (38). Since ADA2 colocalizes with the lysosomal marker LAMP 2, it is likely sorted into the lysosomal pathway via the mannose-6-phosphate dependent pathway (38). There is no contradiction between the presence of ADA2 in plasma, secretion by macrophages and a lysosomal location, since lysosomal proteins are normally localized in the serum at low concentrations (43) and are secreted by cultured macrophages (44). There is no contradiction either that recombinant ADA2 in overexpressing cells are secreted (10), as recombinant soluble lysosomal proteins are mainly secreted in cell cultures (45). However, the elevated levels of plasma ADA2 detected in patients with chronic inflammations, such as HIV, rheumatoid arthritis, empyema, tuberculosis, sarcoidosis, chronic hepatitis and liver cirrhosis (46–52) cannot be reconciled with a strictly lysosomal function. The ADA2 secretion seems to originate from monocyte/macrophage lineages (53), and although the secretion of ADA2 appears low in unstimulated monocytes, upon stimulation of human peripheral blood monocytes with the calcium ionophore A23187 the secretion of ADA2 increased substantially (54). A simple explanation is that ADA2 is stored in Ca^2+^-sensitive secretory vesicles. The recent finding that ADA2 is sorted into the lysosomal pathway (38, 39), suggests that ADA2 may be subject to regulated secretion via the endosomes, as increases in cytosolic calcium may lead to exocytosis of lysosome-like vesicles (55), and since Ca^2+^-induced exocytosis in immune cells can be stimulated by danger signals (56). Macrophages have the capacity to rapidly secrete a wide range of inflammatory mediators that influence the development and extent of an inflammatory response. However, there is a common misconception that macrophages lack secretory lysosomes that enable the rapid release of inflammatory mediators upon signaling (57). For example, MHC class II molecules are stored in the macrophages within a compartment with characteristics of a secretory lysosome (58). Cells that have secretory lysosomes package both lysosomal and secretory proteins in one compartment, which has been named “late endosomes” to be distinguished from “mature lysosomes” (59). Thus, ADA2 may be sorted from late endosomes into the extracellular space via a regulated pathway conforming to the increased concentration of plasma ADA2 during chronic inflammation. MAS (macrophage activation syndrome) is a rare complication of viral infections causing a devastating cytokine storm. The concomitantly high increase of ADA2 in serum (52, 60), suggests that proinflammatory cytokines may contribute to the exocytosis of vesicles containing ADA2. The mannose-6-phosphate dependent lysosomal sorting of ADA2 does not exclude secretion, as granzyme A and B in cytotoxic T-cells require mannose-6-phosphorylated to be sorted into secretory lysosomes (61), from where they are exocytosed upon cell stimulation in a Ca^2+^-dependent manner (62). The study of a familial form of hemophagocytic lymphohistiocytosis (FHL) due to deficiency of perforin has uncovered some of the proteins involved in the vesicular transport of secretory lysosomes, as Rab27a, Munc13-4, Syntaxin11, and Munc18-2 (63). As ADA2 isolated from tissues contain mainly mannose-terminal N-glycans (Figure 2), secreted ADA2 may be quickly endocytosed via the mannose receptor on macrophages. Thus, activated macrophages may secrete larger amounts of ADA2 during inflammation than evaluated by the increase in plasma concentration due to a rapid clearance via the mannose receptor (64). This uptake will bring ADA2 and any binding partner, like for example an oligonucleotide complex, into mature lysosomes where ADA2 may take part in the lysosomal oligonucleotide metabolism. Thus, ADA2 by utilizing a well-known pathway for regulated secretion of immune-modulatory proteins stored in secretory lysosomes, may accommodate both a secretory and a lysosomal role.

**Figure 2:**
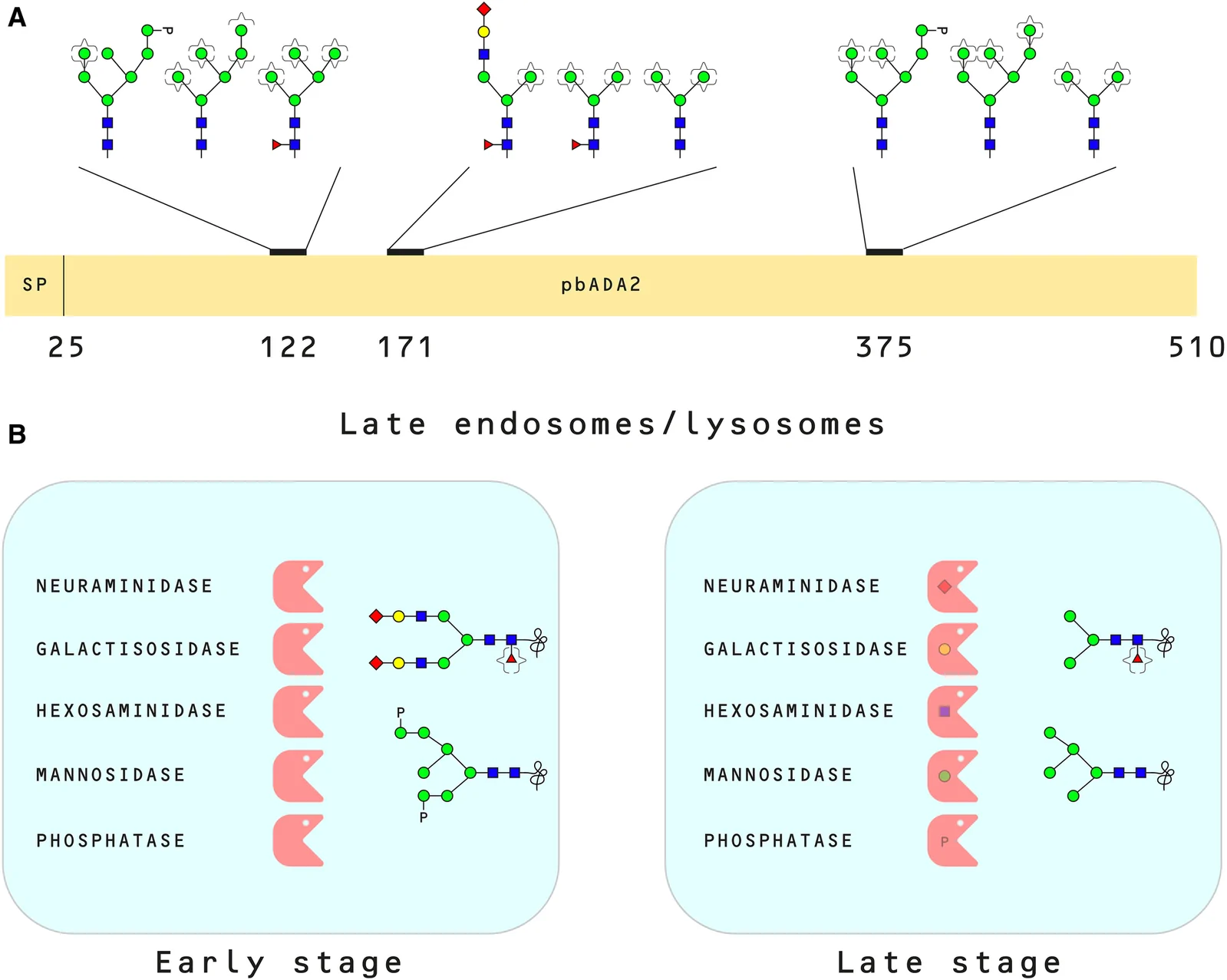
**(A)** the main N-linked glycan structures linked to each of the three N-glycosylation sites of porcine brain (pb) ADA2 (37), showing that the terminal sugars consist mainly of mannose-residues (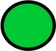). **(B)** Predicted mechanism of N-glycan trimming resulting in the glycan structures depicted in A. When nascent ADA2 enters late endosomes/lysosomes from trans Golgi the N-glycans in ADA2 is untrimmed, consisting of mainly mannose-6-phosphorylated and complex type structures (left). As the acid exoglycosidases and phosphatases of late endosomes/lysosomes hydrolyze the N-glycans, the mannose-6-phosphorylated structures are trimmed into oligomannosidic structures and complex type structures trimmed into paucimannose type structures (right), reminiscent of those structures found in porcine brain ADA2 **(A)**. Symbols: 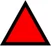
=Fuc, 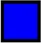
= GlcNAc, 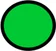
=Man, 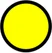
=Gal, 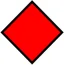
=Neu, P = phosphate.

## Cytokines in DADA2

Most autoinflammatory syndromes cause activation of immune pathways that lead to production of key cytokines as interleukin-1, tumor necrosis factor alpha (TNF) and type 1 interferons (IFN-1) (65). Early studies identified TNF and IFN-1 as the key drivers of inflammation in DADA2.

A) **Type 1 interferon**

Type 1 interferons are classically involved in the response to viral infections. Autoinflammatory conditions with overactivation of IFN-1, known as interferonopathies, encompass a clinical spectrum including small vessel vasculitis and a wide range of autoimmune manifestations (66). Since the initial description of the prototypical interferonopathy, Aicardi-Goutieres syndrome (AGS), several monogenic defects involved in the nucleic acid metabolism has been added to this disease group (67). There are clinical overlaps between DADA2 and interferonopathies, as cutaneous small vessel vasculitis and variable cytopenia and autoantibodies as seen in AGS and SAVI (67, 68). However, the tendency to cause ischemic and hemorrhagic infarct and neutrophil activation are unique features in DADA2 (11). Moreover, TNF inhibitor treatment is not effective in the treatment of AGS or other interferonopathies (69, 70), whereas it is the first line choice of treatment for vasculitis-DADA2 (23, 24). Whereas the contribution of TNF to the pathophysiology of DADA2 is unquestionable, as TNF inhibitor reverses several of the vasculitis-related phenotypes, the contribution of IFN-1 has remained unclear. IFN-1 may contribute primarily to the bone marrow defect, as this phenotype cannot be treated by TNF inhibitors, and since IFN-1 is a potent inhibitor of hematopoietic cell proliferation and may cause aplastic anemia (71), reminiscent of pure red cell aplasia of DADA2. A type I interferonopathy due to DNase II deficiency in three children were recently described (61). All three children presented in the neonatal period with life-threatening anemia and thrombocytopenia. A similar hematological involvement was found in patients with pseudo-infection syndromes due to loss-of-function mutations in *TREX1* (72) and *USP18* (73). Taking into consideration the type 1 interferonopathy and severe anemia in DNase knock out mice (74) there appears to be a sensitivity of both murine and human erythropoiesis to high production of type I interferon due to abnormal lysosomal oligonucleotide metabolism. As DADA2 is an interferonopathy a lysosomal function in the oligonucleotide metabolism of ADA2 may provide an explanation of the bone marrow involvement in DADA2.

B) **Tumor necrosis factor**

TNF is recognized as a central cytokine in the pathogenesis of DADA2 due to the favorable clinical response to TNF inhibitors. Many studies have found significant levels of TNF in the peripheral blood and biopsy tissues from patients (10, 13–76) and gene signatures of TNF signaling (75, 76). TNF has profound effect on the vasculature, by causing remodeling of endothelial cell morphology and endothelial barrier disruption (77), reminiscent of the vascular phenotype of DADA2. The source of TNF is likely proinflammatory M1 macrophages with an increased capacity to produce TNF upon stimulation *in vitro (*37). ADA2 may promote macrophage differentiation into M2 macrophages in glioblastoma (78), leading to the hypothesis that ADA2 acts as an M1/M2 switch, where a shift towards M1 macrophages leads to the vasculitis phenotype of DADA2. Carmona et al. observed that coculture of neutrophil extracellular traps (NETs) isolated from DADA2 patients with macrophages caused an increased TNF production in macrophages (26). As DADA2 patients exhibit neutrophil signatures (12) and an increased number of low-density granulocytes (LDGs) that are prone to spontaneous NETosis (26), activated neutrophils may secrete proinflammatory molecules or interact directly with macrophages resulting in TNF-release from macrophages (26). Neutrophils were activated by adenosine via binding to adenosine receptors, conforming to a significant increase in plasma adenosine during inflammation (26). The activation was prevented by recombinant ADA2 *in vitro*, but not ADA1 suggesting that the mechanism does not involve adenosine deamination. These results led to a hypothesis that proinflammatory metabolites generated in an inflamed area activate neutrophils resulting in a switch towards M1 macrophages, causing secretion of TNF and remodeling of endothelial cells. This chain of events may cause further release of proinflammatory mediators resulting in sustained neutrophil activation and inflammation. ADA2 by inactivating or clearing these metabolites may prevent neutrophil activation and thus slow down the vicious circuit. This model conforms to a regulated secretion of ADA2 where ADA2 is secreted due to a danger signal, as the protein is only required in the circulation during inflammation. A putative target for ADA2 is circulatory oligonucleotide complexes as they increase in inflamed areas due to NETosis and apoptosis. If these complexes are not cleared from circulation they may be hydrolyzed causing the release of adenosine and other proinflammatory mediators.

## Genotype/phenotype correlation

In the most comprehensive study of genotype/phenotype relationships, Lee et al. compared DADA2 patients with hematologic events, including pure red cell aplasia (PRCA) and bone marrow failure (BMF) syndrome with patients affected with vasculitis (79). Although the mutations in both groups were scattered across the ADA2 gene, the types of mutations varied, mainly missense mutations in vasculitis patients while only half of the mutations in the PRCA group were of missense type, the remaining mainly deletions/insertions. All the recombinant proteins with missense mutations of the PRCA group exhibited ADA activities below a threshold of 3%, while in the vasculitis group mainly above 3%, suggesting that only the most detrimental mutations cause hematologic symptoms. Overexpressing systems may result in higher production of active enzymes than *in vivo* conditions, due to oversaturation of the folding control system, and the results should thus be treated with precaution. Indeed, in the serum of DADA2 patients the ADA2 activities are usually below the detection limit, while three recombinant ADA2-variants with the respective missense-mutations, G25C, P344L and A357 T, resulted in ADA-activities about 50% of wild type (79). Even taking this into consideration it seems that detrimental mutations have a higher probability of causing hematologic symptoms. This may justify a future classification of DADA2 into a severe and mild form. A challenge in the classification of DADA2 is phenotypic diversity even among patients affected with the same mutation (79). As this may also occur in twins (80), differences in the genetic background do not adequately explain these phenotypic variations. Environmental factors, such as virus infections may act as triggers.

## Diagnosis and therapy

The broad clinical spectrum of DADA2 and overlap with other diseases of immunodeficiency is a challenge for early diagnosis and treatment. DADA2 is among the common monogenic autoinflammatory diseases, with an estimate of more than 35 000 cases worldwide. An early diagnosis is necessary for early treatment resulting in improved quality of life. Recently, a DADA2 Consensus Committee developed consensus statements for diagnostic screening, clinical and laboratory evaluation, and management of DADA2 based on disease phenotype (81). Diagnostic testing is mainly measured by the EHNA-independent adenosine deaminase activity in plasma, and sequencing of the ADA2 gene (81). As free adenosine may not be the natural substrate of ADA2 future studies may reveal cases of DADA2 where the ADA-activity is high although ADA2 is dysfunctional. With increased understanding the diagnostic tools may also improve.

Tumor necrosis factor inhibitor (TNFi) treatment for DADA2 is effective for vascular symptoms, but it is ineffective for bone marrow manifestations such as pure red cell aplasia. Anti-drug antibodies are the most used TNFi, but may cause allergic reactions or loss of drug efficacy, requiring switching and long term complications. Hematopoietic stem cell transplant (HSCT) appears to be curative of DADA2 based on experience from 30 patients (21). Lentovirus delivery of an ADA2 construct into DADA2 macrophages restored normal levels of inflammatory cytokines *in vitro* (37) and recently revealed long-term engraftment and multilineage reconstitution in mouse (82, 83). These findings suggest that ADA2 gene therapy may be a future alternative to allogeneic HSCT.

## Future perspectives

Much remains to decipher the mechanisms that underly this complex disease. Due to the ever-increasing number of novel DADA2 mutations and clinical studies there is a strong fundament for unraveling the physiological function of ADA2 and the molecular mechanisms leading to disease. The aim of this review is to propose a novel direction of ADA2 research based on a framework that ADA2 is both a regulated secretory and a lysosomal protein involved in oligonucleotide metabolism and with a vital function in both compartments. Future studies should focus on revealing the mechanism of the regulated secretion of ADA2 in macrophages. The recent discovery that ADA2 binds and modifies synthetic oligonucleotides *in vitro* should be extended to investigate how ADA2 binds oligonucleotides during *in vivo* conditions, what types of oligonucleotides are binding, and how the oligonucleotides are modified by ADA2. A function of ADA2 in oligonucleotide metabolism of lysosomes may provide an explanation to the interferonopathy of DADA2 as several diseases connected to abnormal oligonucleotide metabolism are linked to interferonopathies, such as for example DNase 2 deficiency. The anemia and thrombocytopenia of DNase 2 deficiency may be related to the hematological type of DADA2. Interestingly TNF treatment is inefficient towards DNase 2 deficiency (70), reminiscent of the hematological type of DADA2. Thus, the expression of IFN-1 and TNF in DADA2 may be a result of two different forms of ADA2, the lysosomal and the extracellular, harboring different functions. Thus, the studies on the properties of the ADA2 protein should differentiate between the extracellular homodimer form and the lysosomal form.

## Conclusion

Despite tremendous progress for the last 12 years, the knowledge of the physiological function of ADA2 and the molecular mechanisms leading to disease have remained incomplete. In this review we have discussed how a lysosomal role of ADA2 can coexist with an extracellular function, and how the role of ADA2 in oligonucleotide metabolism may explain many aspects of the molecular pathophysiology of DADA2. Although our model provides explanations, it has yet to be experimentally tested. For example, the binding of ADA2 to oligonucleotides was studied *in vitro* on artificial oligonucleotides and has not yet been demonstrated *in vivo* on physiological oligonucleotides. The secretory route of ADA2 has not yet been experimentally investigated in detail and the physiological role of ADA2 in the lysosomes is insufficiently known.

## Data Availability

The original contributions presented in the study are included in the article/Supplementary Material, further inquiries can be directed to the corresponding author.

## References

[1] Schrader WP Pollara B Meuwissen HJ. Characterization of the residual adenosine deaminating activity in the spleen of a patient with combined immunodeficiency disease and adenosine deaminase deficiency. Proc Natl Acad Sci U S A. (1978) 75:446–50. 10.1073/pnas.75.1.446 24216 PMC411266

[2] Daddona PE Kelley WN. Characteristics of an amidohydrolase distinct from adenosine deaminase in cultured human lymphoblasts. Biochim Biophys Acta. (1981) 658:280–90. 10.1016/0005-2744(81)90298-9 6972784

[3] Homma K Matsushita T Natoru S. Purification, characterization and cDNA cloning of a novel growth factor from the conditioned medium of NIH-sape-4, an embryonic cell line of *Sarcophaga Peregrina* (flesh fly). J Biol Chem. (1996) 271:13770–5. 10.1074/jbc.271.23.13770 8662793

[4] Riazi MA Brinkman-Mills P Nguyen T Pan H Phan S Ying F. The human homolog of insect-derived growth factor, CECR1, is a candidate gene for features of cat eye syndrome. Genomics. (2000) 64:277–85. 10.1006/geno.1999.6099 10756095

[5] Zavialov AV Engström A. Human ADA2 belongs to a new family of growth factors with adenosine deaminase activity. Biochem J. (2005) 391:51–7. 10.1042/BJ20050683 15926889 PMC1237138

[6] Zavialov AV Yu X Spillmann D Lauvau G Zavialov AV. Structural basis for the growth factor activity of human adenosine deaminase ADA2. J Biol Chem. (2010) 285:12367–77. 10.1074/jbc.M109.083527 20147294 PMC2852975

[7] Homma KJ Tanaka Y Matsushita T Yokoyama K Matsui H Natori S. Adenosine deaminase activity of insect-derived growth factor is essential for its growth factor activity. J Biol Chem. (2001) 276:43761–6. 10.1074/jbc.M105088200 11562360

[8] Zurovec M Dolezal T Gazi M Pavlova E Bryant PJ. Adenosine deaminase-related growth factors stimulate cell proliferation in Drosophila by depleting extracellular adenosine. Proc Natl Acad Sci U S A. (2002) 99:4403–8. 10.1073/pnas.062059699 11904370 PMC123660

[9] Tarrant TK Kelly SJ Hershfield MS. Elucidating the pathogenesis of adenosine deaminase 2 deficiency: current status and unmet needs. Exp Opin on Orphan Drugs. (2021) 9:257–64. 10.1080/21678707.2021.2050367

[10] Zhou Q Yang D Ombrello AK Zavialov AV Toro C Zavialov AV. Early-onset stroke and vasculopathy associated with mutations in ADA2. N Engl J Med. (2014) 370:911–20. 10.1056/NEJMoa1307361 24552284 PMC4193683

[11] Navon Elkan P Pierce SB Segel R Walsh T Barash J Padeh S. Mutant adenosine deaminase 2 in a polyarteritis nodosa vasculopathy. N Engl J Med. (2014) 370:921–31. 10.1056/NEJMoa1307362 24552285

[12] Belot A Wassmer E Twilt M Lega J-C Zeef LAH Oojageer A. Mutations in CECR1 associated with a neutrophil signature in peripheral blood. Pediatr Rheumatol Online J. (2014) 12:44. 10.1186/1546-0096-12-44 25278816 PMC4181355

[13] Caorsi R Penco F Grossi A Insalaco A Omenetti A Alessio M. ADA2 deficiency (DADA2) as an unrecognised cause of early onset polyarteritis nodosa and stroke: a multicentre national study. Ann Rheum Dis. (2017) 76:1648–56. 10.1136/annrheumdis-2016-210802 28522451

[14] Chong-Neto HJ Segundo GRS Bandeira M Chong-Silva DC Rosário CS Riedi CA. Homozygous splice ADA2 gene mutation causing ADA-2 deficiency. J Clin Immunol. (2019) 39:842–5. 10.1007/s10875-019-00697-2 31617030

[15] Gibson KM Morishita KA Dancey P Moorehead P Drögemöller B Han X. Identification of novel adenosine deaminase 2 gene variants and varied clinical phenotype in pediatric vasculitis. Arthritis Rheumatol. (2019) 71:1747–55. 10.1002/art.40913 31008556

[16] Nanthapisal S Murphy C Omoyinmi E Hong Y Standing A Berg S. Deficiency of adenosine deaminase type 2: a description of phenotype and genotype in fifteen cases. Arthritis Rheumatol. (2016) 68:2314–22. 10.1002/art.39699 27059682

[17] Özen S Batu ED Taşkıran EZ Özkara HA Ünal Ş Güleray N. A monogenic disease with a variety of phenotypes: deficiency of adenosine deaminase 2. J Rheumatol. (2020) 47:117–25. 10.3899/jrheum.181384 31043544

[18] Tanatar A Karadağ ŞG Sözeri B Sönmez HE Çakan M Kendir Demirkol Y. ADA2 deficiency: case series of five patients with varying phenotypes. J Clin Immunol. (2020) 40:253–8. 10.1007/s10875-019-00734-0 31848804

[19] Escherich C Bötticher B Harmsen S Hömberg M Schaper J Lorenz MR. The growing spectrum of DADA2 manifestations-diagnostic and therapeutic challenges revisited. Front Pediatr. (2022) 10:885893. 10.3389/fped.2022.885893 35774100 PMC9237362

[20] Bulté D Barzaghi F Mesa-Nuñez C Rigamonti C Basso-Ricci L Visconti C. Early bone marrow alterations in patients with adenosine deaminase 2 deficiency across disease phenotypes and severities. J Allergy Clin Immunol. (2025) 155:616–627e8. 10.1016/j.jaci.2024.09.007 PMC11804788 39284370

[21] Hashem H Bucciol G Ozen S Unal S Bozkaya IO Akarsu N. Hematopoietic cell transplantation cures adenosine deaminase 2 deficiency: report on 30 patients. J Clin Immunol. (2022) 42:1580–1. 10.1007/s10875-022-01280-y 35499644 PMC9674743

[22] Van Eyck L Hershfield MS Pombal D Kelly SJ Ganson NJ Moens L. Hematopoietic stem cell transplantation rescues the immunologic phenotype and prevents vasculopathy in patients with adenosine deaminase 2 deficiency. J Allergy Clin Immunol. (2015) 135:283–7. 10.1016/j.jaci.2014.10.010 25457153 PMC4282724

[23] Ombrello AK Qin J Hoffmann PM Kumar P Stone D Jones A. Treatment strategies for deficiency of adenosine deaminase 2. N Engl J Med. (2019) 380:1582–4. 10.1056/NEJMc1801927 30995379 PMC7372950

[24] Maccora I Maniscalco V Campani S Carrera S Abbati G Marrani E. A wide spectrum of phenotype of deficiency of deaminase 2 (DADA2): a systematic literature review. Orphanet J Rare Dis. (2023) 18:117. 10.1186/s13023-023-02721-6 37179309 PMC10183141

[25] Cooray S Omyinmi E Hong Y Papadopoulou C Harper L Al-Abadi E. Anti-tumour necrosis factor treatment for the prevention of ischaemic events in patients with deficiency of adenosine deaminase 2 (DADA2). Rheumatology. (2021) 60:4373–8. 10.1093/rheumatology/keaa837 33420503

[26] Carmona-Rivera C Khaznadar SS Shwin KW Irizarry-Caro JA O’Neil LJ Liu Y. Deficiency of adenosine deaminase 2 triggers adenosine-mediated NETosis and TNF production in patients with DADA2. Blood. (2019) 134:395–406. 10.1182/blood.2018892752 31015188 PMC6659253

[27] Kaljas Y Liu C Skaldin M Wu C Zhou Q Lu Y. Human adenosine deaminases ADA1 and ADA2 bind to different subsets of immune cells. Cell Mol Life Sci. (2017) 74:555–70. 10.1007/s00018-016-2357-0 27663683 PMC11107696

[28] Zavialov AV Gracia E Glaichenhaus N Franco R Zavialov AV Lauvau G. Human adenosine deaminase 2 induces differentiation of monocytes into macrophages and stimulates proliferation of T helper cells and macrophages. J Leukocyte Biol. (2010) 88:279–90. 10.1189/jlb.1109764 20453107

[29] Dhanwani R Takahashi M Mathews IT Lenzi C Romanov A Watrous JD. Cellular sensing of extracellular purine nucleosides triggers an innate IFN-*β* response. Sci Adv. (2020) 6:eaba3688. 10.1126/sciadv.aba3688 32743071 PMC7375821

[30] Plagemann PG Wohlhueter RM Kraupp M. Adenosine uptake, transport, and metabolism in human erythrocytes. J Cell Physiol. (1985) 125:330–6. 10.1002/jcp.1041250223 3877060

[31] Iijima R Kunieda T Yamaguchi S Kamigaki H Fujii-Taira I Sekimizu K. The extracellular adenosine deaminase growth factor, ADGF/CECR1, plays a role in xenopus embryogenesis via the adenosine/P1 receptor. J Biol Chem. (2008) 283:2255–64. 10.1074/jbc.M709279200 18032387

[32] Daddona PE Kelley WN. Characteristics of an aminohydrolase distinct from adenosine deaminase in cultured human lymphoblasts. Biochim Biophys Acta. (1981) 658:280–90. 10.1016/0005-2744(81)90298-9 6972784

[33] Ratech H Hirschhorn R. Serum adenosine deaminase in normals and in a patient with adenosine deaminase deficient-severe combined immunodeficiency. Clin Chim Acta. (1981) 115:341–7. 10.1016/0009-8981(81)90247-3 6975191

[34] Iwaki-Egawa S Namiki C Watanabe Y. Adenosine deaminase 2 from chicken liver: purification, characterization, and N-terminal amino acid sequence. Comp Biochem Physiol Part B: Biochem Mol Biol. (2004) 137:247–54. 10.1016/j.cbpc.2003.11.010 14990221

[35] Charlab R Rowton ED Ribeiro JM. The salivary adenosine deaminase from the sand fly lutzomyia longipalpis. Exp Parasitol. (2000) 95:45–53. 10.1006/expr.2000.4503 10864517

[36] Tzertzinis G Ganatra MB Ruse C Taron CH Causey B Wang L. The AMP deaminase of the mollusk helix pomatia is an unexpected member of the adenosine deaminase-related growth factor (ADGF) family. PLoS One. (2023) 18:e0286435. 10.1371/journal.pone.0286435 37471401 PMC10358891

[37] Zoccolillo M Brigida I Barzaghi F Scala S Hernández RJ Basso-Ricci L. Lentiviral correction of enzymatic activity restrains macrophage inflammation in adenosine deaminase 2 deficiency. Blood Adv. (2021) 5:3174–87. 10.1182/bloodadvances.2020003811 34424322 PMC8405196

[38] Greiner-Tollersrud OK Krausz M Boehler V Polyzou A Seidl M Spahiu A. ADA2 is a lysosomal deoxyadenosine deaminase acting on DNA involved in regulating TLR9-mediated immune sensing of DNA. Cell Rep. (2024) 43:114899. 10.1016/j.celrep.2024.114899 39441717

[39] Dong L Luo W Maksym S Robson SC Zavialov AV. Adenosine deaminase 2 regulates the activation of the toll-like receptor 9 in response to nucleic acids. Front Med. (2024) 18:814–30. 10.1007/s11684-024-1067-5 39078537

[40] Greiner-Tollersrud OK Krausz M Boehler V Polyzou A Seidl M Spahiu A. ADA2 is a lysosomal DNase regulating the type-I interferon response. bioRxiv. (2020). 10.1101/2020.06.21.162990

[41] Insalaco A Moneta GM Pardeo M Caiello I Messia V Bracaglia C. Variable clinical phenotypes and relation of interferon signature with disease activity in ADA2 deficiency. J Rheumat. (2019) 46:523–6. 10.3899/jrheum.180045 30647181

[42] Skrabl-Baumgartner A Plecko B Schmidt WM Konig N Hershfield M Gruber-Sedlmayr U. Autoimmune phenotype with type I interferon signature in two brothers with ADA2 deficiency carrying a novel *CECR1* mutation. Pediat Rheumat Online J. (2017) 15:67. 10.1186/s12969-017-0193 PMC5568374 28830446

[43] Lombardo A Caimi L Marchesini S Goi GC Tettamanti G. Enzymes of lysosomal origin in human plasma and serum: assay conditions and parameters influencing the assay. Clin Chim Acta. (1980) 108:337–46. 10.1016/0009-8981(80)90339-3 6258826

[44] Schnyder J Baggiolini M. Secretion of lysosomal hydrolases by stimulated and nonstimulated macrophages. J Exp Med. (1978) 148:435–50. 10.1084/jem.148.2.435 29935 PMC2184949

[45] Ioannou YA Bishop DF Desnick RJ. Overexpression of human alpha-galactosidase A results in its intracellular aggregation, crystallization in lysosomes, and selective secretion. J Cell Biol. (1992) 119:1137–50. 10.1083/jcb.119.5.1137 1332979 PMC2289730

[46] Niedzwicki JG Mayer KH Abushanab E Abernethy DR. Plasma adenosine deaminase2 is a marker for human immunodeficiency virus-1 seroconversion. Am J Hematol. (1991) 37:152–5. 10.1002/ajh.2830370303 1858768

[47] Tsuboi I Sagawa K Shichijo S Yokoyama MM Ou DW Wiederhold MD. Adenosine deaminase isoenzyme levels in patients with human T-cell lymphotropic virus type 1 and human immunodeficiency virus type 1 infections. Clin Diagn Lab Immunol. (1995) 2:626–30. 10.1128/cdli.2.5.626-630.1995 8548545 PMC170210

[48] Kobayashi F Ikeda T Marumo F Sato C. Adenosine deaminase isoenzymes in liver disease. Am J Gastroenterol. (1993) 88:266–71. PMID: 8424431 8424431

[49] Pérez-Rodriguez E Jiménez Castro D. The use of adenosine deaminase and adenosine deaminase isoenzymes in the diagnosis of tuberculous pleuritis. Curr Opin Pulm Med. (2000) 6:259–66. 10.1097/00063198-200007000-00002 10912630

[50] Iwaki-Egawa S Watanabe Y Matsuno H. Correlations between matrix metalloproteinase-9 and adenosine deaminase isozymes in synovial fluid from patients with rheumatoid arthritis. J Rheumatol. (2001) 28:485–9. PMID: 11296946 11296946

[51] Porcel JM. Tuberculous pleural effusion. Lung. (2009) 187:263–70. 10.1007/s00408-009-9165-3 19672657

[52] Lim J Doss J Shen J Hershfield M Tarrant T. A review of adenosine deaminase 2 (ADA2) as a biomarker of monocyte/macrophage activation. Curr Allergy Asthma Rep. (2026) 26:2. 10.1007/s11882-025-01249-y PMC12764692 41484495

[53] Karlsson M Zhang C Méar L Zhong W Digre A Katona B. A single-cell type transcriptomics map of human tissues. Sci Adv. (2021) 7:eabh2169. 10.1126/sciadv.abh2169 34321199 PMC8318366

[54] Iwaki-Egawa S Yamamoto T Watanabe Y. Human plasma adenosine deaminase 2 is secreted by activated monocytes. Biol Chem. (2006) 387:319–21. 10.1515/BC.2006.042 16542154

[55] Jaiswal JK Andrews NW Simon SM. Membrane proximal lysosomes are the major vesicles responsible for calcium-dependent exocytosis in nonsecretory cells. J Cell Biol. (2002) 159:625–35. 10.1083/jcb.200208154 12438417 PMC2173094

[56] Ayala BP Vasquez B Clary S Tainer JA Rodland K So M. The pilus-induced Ca2 + flux triggers lysosome exocytosis and increases the amount of Lamp1 accessible to neisseria IgA1 protease. Cell Microbiol. (2001) 3:265–75. 10.1046/j.1462-5822.2001.00112.x 11298650

[57] Murray RZ Stow JL. Cytokine secretion in macrophages: sNAREs, rabs, and membrane trafficking. Front Immunol. (2014) 27:538. 10.3389/fimmu.2014.00538 PMC4209870 25386181

[58] Peters PJ Neefjes JJ Oorschot V Ploegh HL Geuze HJ. Segregation of MHC class II molecules from MHC class I molecules in the Golgi complex for transport to lysosomal compartments. Nature. (1991) 349:669–76. 10.1038/349669a0 1847504

[59] Claus V Jahraus A Tjelle T Berg T Kirschke H Faulstich H. Lysosomal enzyme trafficking between phagosomes, endosomes, and lysosomes in J774 macrophages. Enrichment of cathepsin H in early endosomes.. J Biol Chem. (1998) 273:9842–51. 10.1074/jbc.273.16.9842 9545324

[60] Lee PY Schulert GS Canna SW Huang Y Sundel J Li Y. Adenosine deaminase 2 as a biomarker of macrophage activation syndrome in systemic juvenile idiopathic arthritis. Ann Rheum Dis. (2020) 79:225–31. 10.1136/annrheumdis-2019-216030 31707357 PMC7385992

[61] Griffiths GM Isaaz S. Granzymes A and B are targeted to the lytic granules of lymphocytes by the mannose-6-phosphate receptor. J Cell Biol. (1993) 120:885–96. 10.1083/jcb.120.4.885 8432729 PMC2200067

[62] Velotti F MacDonald HR Nabholz M. Granzyme A secretion by normal activated lyt-2 + and L3T4+ T cells in response to antigenic stimulation. Eur J Immunol. (1987) 17:1095–9. 10.1002/eji.1830170804 2957213

[63] Griffiths GM. Secretion from myeloid cells: secretory lysosomes. Microbiol Spectr. (2016) 4:4. 10.1128/microbiolspec.MCHD-0030-2016 27726815

[64] Cummings RD. The mannose receptor ligands and the macrophage glycome. Curr Opin Struct Biol. (2022) 75:102394. 10.1016/j.sbi.2022.102394 35617912 PMC10243190

[65] Nigrovic PA Lee PY Hoffman HM. Monogenic autoinflammatory disorders: conceptual overview, phenotype, and clinical approach. J Allergy Clin Immunol. (2020) 146:925–37. 10.1016/j.jaci.2020.08.017 33160483 PMC7727443

[66] Volpi S Picco P Caorsi R Candotti F Gattorno M. Type I interferonopathies in pediatric rheumatology. Pediatr Rheumatol Online J. (2016 Jun 4) 14(1):35. 10.1186/s12969-016-0094-4 PMC4893274 27260006

[67] Crow YJ Manel N. Aicardi-goutières syndrome and the type I interferonopathies. Nat Rev Immunol. (2015) 15:429–40. 10.1038/nri3850 26052098

[68] Liu Y Jesus AA Marrero B Yang D Ramsey SE Montealegre Sanchez GA. Activated STING in a vascular and pulmonary syndrome. N Engl J Med. (2014) 371:507–18. 10.1056/NEJMoa1312625 25029335 PMC4174543

[69] Takanohashi A Prust M Wang J Gordish-Dressman H Bloom M Rice GI. Elevation of proinflammatory cytokines in patients with aicardi-goutières syndrome. Neurology. (2013) 80:997–1002. 10.1212/WNL.0b013e3182872694 23408864 PMC3653200

[70] Rodero MP Tesser A Bartok E Rice GI Della Mina E Depp M. Type I interferon-mediated autoinflammation due to DNase II deficiency. Nat Commun. (2017) 8:2176. 10.1038/s41467-017-01932-3 29259162 PMC5736616

[71] Zoumbos NC Gascon P Djeu JY Young NS. Interferon is a mediator of hematopoietic suppression in aplastic anemia *in vitro* and possibly *in vivo*. Proc Natl Acad Sci USA. (1985) 82:188–92. 10.1073/pnas.82.1.188 3918301 PMC396997

[72] Rice G Patrick T Parmar R Taylor CF Aeby A Aicardi J. Clinical and molecular phenotype of aicardi-goutieres syndrome. Am J Hum Genet. (2007) 81:713–25. 10.1086/521373 17846997 PMC2227922

[73] Meuwissen MEC Schot R Buta S Oudesluijs G Tinschert S Speer SD. Human USP18 deficiency underlies type 1 interferonopathy leading to severe pseudo-TORCH syndrome. J Exp Med. (2016) 213:1163–74. 10.1084/jem.20151529 27325888 PMC4925017

[74] Yoshida H Okabe Y Kawane K Fukuyama H Nagata S. Lethal anemia caused by interferon-beta produced in mouse embryos carrying undigested DNA. Nat. Immunol. (2005) 6:49–56. 10.1038/ni1146 15568025

[75] Watanabe N Gao S Wu Z Batchu S Kajigaya S Diamond C. Analysis of deficiency of adenosine deaminase 2 pathogenesis based on single-cell RNA sequencing of monocytes. J Leukoc Biol. (2021) 110:409–24. 10.1002/JLB.3HI0220-119RR 33988272

[76] Deuitch NT Yang D Lee PY Yu X Moura NS Schnappauf O. TNF inhibition in vasculitis management in adenosine deaminase 2 deficiency (DADA2). J Allergy Clin Immunol. (2022) 149:1812–16. 10.1016/j.jaci.2021.10.030 34780847

[77] Marcos-Ramiro B García-Weber D Millán J. TNF-induced endothelial barrier disruption: beyond actin and rho. Thromb Haemost. (2014) 112:1088–102. 10.1160/th14-04-0299 25078148

[78] Zhu C Chrifi I Mustafa D Van Der Weiden M Leenen PJM Duncker DJ. CECR1-mediated cross talk between macrophages and vascular mural cells promotes neovascularization in malignant glioma. Oncogene. (2017) 36:5356–68. 10.1038/onc.2017.145 28534507 PMC5611481

[79] Lee PY Kellner ES Huang Y Furutani E Huang Z Bainter W. Genotype and functional correlates of disease phenotype in deficiency of adenosine deaminase 2 (DADA2). J Allergy Clin Immunol. (2020) 145:1664–72.e10. 10.1016/j.jaci.2019.12.908 31945408 PMC7282972

[80] Aksu MD Özen S Aksu T Gürel A Çetinkaya A Ünal Ş. Ünal Ş. Extreme phenotypic variation in siblings with identical homozygous mutations causing ADA2 deficiency: a case series. Turk J Haematol. (2025) 42:61–4. 10.4274/tjh.galenos.2025.2024.0373 39764790 PMC11869147

[81] Lee PY Davidson BA Abraham RS Alter B Arostegui JI Bell K. Evaluation and management of deficiency of adenosine deaminase 2: an international consensus statement. JAMA Netw Open. (2023) 6:e2315894. 10.1001/jamanetworkopen.2023.15894 37256629

[82] Hong Y Burleigh A Liao A Yeung J Bian Y Sebire N. Preclinical evaluation of lentiviral gene therapy for adenosine deaminase 2 deficiency (DADA2): engraftment efficiency and biodistribution in humanised NBSGW mice. Gene Ther. (2025) 32:664–71. 10.1038/s41434-025-00547-4 40555830 PMC12714571

[83] Rigamonti C Bulté D Barzaghi F Mesa-Nuñez C Basso-Ricci L Pettinato E. Gene therapy supports long-term reconstitution of patient hematopoietic stem cells in deficiency of adenosine deaminase 2. Mol Ther Methods Clin Dev. (2025) 33:101624. 10.1016/j.omtm.2025.101624 41321395 PMC12662005

